# Spatiotemporal Dynamics of Scrub Typhus in Jiangxi Province, China, from 2006 to 2018

**DOI:** 10.3390/ijerph18094599

**Published:** 2021-04-26

**Authors:** Shu Yang, Xiaobo Liu, Yuan Gao, Baizhou Chen, Liang Lu, Weiqing Zheng, Renlong Fu, Chenying Yuan, Qiyong Liu, Guichang Li, Haiying Chen

**Affiliations:** 1 The Collaboration Unit for Field Epidemiology of State Key Laboratory of Infectious Disease Prevention and Control, Nanchang Center for Disease Control and Prevention, Nanchang 330038, China; younsoo@163.com (S.Y.); zhengweiqing2001@163.com (W.Z.); renlongzh0829@163.com (R.F.); younsoo@126.com (C.Y.); 2 State Key Laboratory of Infectious Disease Prevention and Control, National Institute for Communicable Disease Control and Prevention, Chinese Center for Disease Control and Prevention, Beijing 102206, China; liuxiaobo@icdc.cn (X.L.); gaoyuancdc@126.com (Y.G.); luliang@icdc.cn (L.L.); liuqiyong@icdc.cn (Q.L.); 3 School of Geography and Information Engineering, China University of Geosciences, Wuhan 430078, China; wuhancbz@tom.com

**Keywords:** scrub typhus, spatiotemporal analysis

## Abstract

*Background*: Scrub typhus (ST) has become a significant potential threat to public health in Jiangxi. Further investigation is essential for the control and management of the spatiotemporal patterns of the disease. *Methods*: Time-series analyses, spatial distribution analyses, spatial autocorrelation analysis, and space-time scan statistics were performed to detect spatiotemporal dynamics distribution of the incidence of ST. *Results*: From 2006 to 2018, a total of 5508 ST cases occurred in Jiangxi, covering 79 counties. The number of ST cases increased continuously from 2006 to 2018, and there was obvious seasonality during the variation process in each year, with a primary peak in autumn (September to October) and a smaller peak in summer (June to August). From 2007 to 2018, the spatial distribution of the ST epidemic was significant heterogeneity, and Nanfeng, Huichang, Xunwu, Anyuan, Longnan, and Xinfeng were hotspots. Seven spatiotemporal clusters were observed using Kulldorff’s space-time scan statistic, and the most likely cluster only included one county, Nanfeng county. The high-risk areas of the disease were in the mountainous, hilly region of Wuyi and the southern mountainous region of Jiangxi. *Conclusions*: Targeted interventions should be executed in high-risk regions for the precise prevention and control of ST.

## 1. Introduction

Scrub typhus (ST) is a neglected life-threatening vector-borne infectious disease. The disease is transmitted by chigger mites (larval trombiculid mites) infected with the gram-negative intracellular rickettsial bacterium *Orientia tsutsugamushi* bacteria [1,2,3]. Symptoms can range from mild (asymptomatic) to lethal and are generally flu-like (fever, headache, myalgia) in their symptomology [4,5]. Rodents, the primary hosts of mites, are indispensable for the survival of chiggers and play a key role in the transmission of ST3 [6,7,8]. There are no long-lasting, broadly protective vaccines available against ST at present [9], and early diagnosis and early treatment can significantly reduce the complications and fatality rate.

The majority of ST cases are reported in the “tsutsugamushi triangle” in the Asia-Pacific area, including, but not limited to, Korea, Japan, China, India, Indonesia, Thailand, Sri Lanka, and the Philippines [10,11]. It is estimated that one million ST cases are found each year within this region, and at least one billion people are at risk of becoming infected [12,13]. Moreover, the incidence in all known endemic regions has begun to rise over the last decade [14,15,16,17]. Recent evidence from China indicated that ST had expanded to all the provinces across both rural and urban areas, and the incidence of ST had increased in an unprecedented manner, in both historically endemic areas, and in new areas without any cases before [18]. Jiangxi province, a relatively new natural focus of the ST outbreak in eastern China, has gradually developed into one of the most seriously affected provinces for the disease [5]. Since the first case of ST was reported in Shanggao county in 1998 [19], the ST epidemic rapidly expanded to 48 counties of Jiangxi by 2016 according to the national ST surveillance data [18]. However, studies on ST in Jiangxi remained limited.

Given that the power of quantitative statistics and mapping visualization, techniques of spatial epidemiological have been widely applied in infectious disease control, prevention, and scientific investigations [20,21,22,23,24]. As a vector-borne infectious disease, the distribution of ST has significant spatial heterogeneity. Understanding the vulnerability of different regions and the potential geographical location of disease outbreaks are helpful for more targeted disease control efforts and assist in the prediction of disease dynamics. Therefore, this study aimed to explore the spatial-temporal patterns of ST in Jiangxi from 2006 to 2018. The results can provide scientific information for public health responses to ST outbreaks.

## 2. Materials and Methods

### 2.1. Study Area

**Figure 1:**
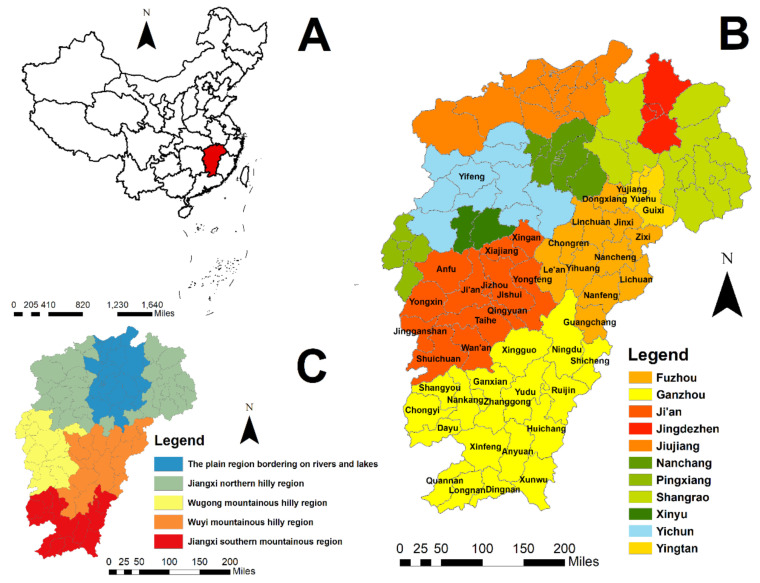
The location of the study area. (**A**) Location of Jiangxi province in China. (**B**) Administrative division of the study area. (**C**) The geographic distribution of five zoographic regions. These maps were generated by ArcGIS software (Version 10.4, ESRI Inc., Redlands, CA, USA; https://www.esri.com/software/arcgis/arcgis-for-desktop, accessed on 11 January 2021).

Jiangxi province (24°29′14″–30°04′44″ N, 113°34′36″–118°28′58″ E) is in eastern China and borders Fujian, Zhejiang, Anhui, Hubei, Hunan, and Guangdong provinces (Figure 1A). It includes 11 cities and 100 counties, with a population of 44.56 million in 2010 and an area of 166,900 km^2^. It has a subtropical monsoon climate, with annual rainfall, annual average temperature, and annual average sunshine ranging from 1341 to 1943 mm, 16.2 to 19.7 °C, 1473 to 2077 h, respectively [25]. Jiangxi is a major agricultural province with an arable land area of around 28,000 km^2^, among which paddy (rice) production covers an area of about 22,000 km^2^ [26] (Figure 1B).

Topographically, this province is dominated by mountains and hills, with basins and valleys spreading over the whole province. Mountains and hills are also the main part of the Jiangnan Hilly Region along the eastern, western, and southern borders. In the central region, hills and valley plains are alternate. The northern portion is the lacustrine alluvial plain of Poyang Lake. More than two-thirds of the province is covered by forests, exceeding the national average of 20% [27]. Through referring to previous studies on the geographic distribution of rodents [21,28], Jiangxi was divided into five zoogeographic regions, including the plain region bordering on rivers and lakes, Jiangxi’s northern hilly region, Wuyi’s mountainous hilly region, Wugong’s mountainous hilly region, and Jiangxi’s southern mountainous region (Figure 1C).

### 2.2. Data Source

ST is a vector-borne notifiable infectious disease. Every medical institution was required to report to the Chinese Center for Disease Control and Prevention through China’s National Statutory Infectious Disease Reporting Information System (CNNDS) (http://www.chinacdc.cn/, accessed on 11 January 2021). The reported information includes age, occupation, onset, and date of onset and diagnosis, case category, and residential address, etc. For this study, the daily data from the ST cases in Jiangxi from January 2006 to December 2018 were extracted from CNNDS. Demographic data at the county level was obtained from the National Bureau of Statistics of the People’s Republic of China. The base map was acquired from the geospatial data cloud (http://www.gscloud.cn/, accessed on 11 January 2021). All cases were geocoded and matched to the corresponding county administrative boundaries using ArcGIS software (Version 10.4, ESRI Inc., Redlands, CA, USA).

ST was diagnosed according to the diagnostic criteria issued by the Ministry of Health of the People’s Republic of China (http://www.nhc.gov.cn/wjw/s9491/200802/38814.shtml, accessed on 11 January 2021). For this study, clinically diagnosed and laboratory-confirmed cases were included, and 105 cases were excluded due to invalid addresses (incomplete, incorrect, or outside study area) or suspected cases.

### 2.3. Data Analysis

A seasonal-trend decomposition of time series analysis was conducted in R software (Version 3.1, AT&T Bell Laboratories, Auckland, New Zealand). A global spatial autocorrelation analysis and a Local Indicators of Spatial Association (LISA) analysis were implemented in ArcGIS software (Version 10.4, ESRI Inc., Redlands, CA, USA) to analyze the spatial patterns and the potential hotspots associated with the ST incidence at the county level. Global Moran’s *I* Index, which ranged from −1 to 1, reflects the similarity of attributes in adjacent spatial regions. Moran’s *I* Index = 0 implied a random spatial distribution. Moran’s *I* Index < 0 suggested a dispersing spatial distribution, and Moran’s *I* Index > 0 implied a clustering spatial distribution. Local Moran’s I for LISA is a measure of the similarity of difference between the attribute of the observation unit and those of surrounding units, and it was calculated to explore significant hot spots (High-High), cold spots (Low-Low), and outliers (High-Low and Low-High) [20]. A Kulldorff’s spatiotemporal scan statistical analysis was used to explore the location of high-risk spatiotemporal clusters by SaTScan Software (Version 9.4, Martin Kulldorff, National Cancer Institute, Bethesda, MD, USA) [29]. The discrete Poisson probability model by a circular window with a radius was employed for monthly data from 2006 to 2018 in the counties. The maximum of the spatial and temporal window sizes was defined as 25% of the at-risk population and 25% of the period [30]. Likelihood ratio tests and Monte Carlo simulations were used to evaluate the significance of the spatiotemporal clusters. A *p*-value of <0.05 was considered to be statistically significant.

The maps were generated in ArcGIS software (Version 10.2, ESRI Inc., Redlands, CA, USA).

### 2.4. Ethics Approval

Permission to conduct this study was approved by the Ethics Review Board of the Nanchang Center for Disease Control and Prevention on 10 June 2020 (No. 2020001). All data analyzed were anonymized.

## 3. Results

### 3.1. Time-Series Analysis for ST

**Figure 2:**
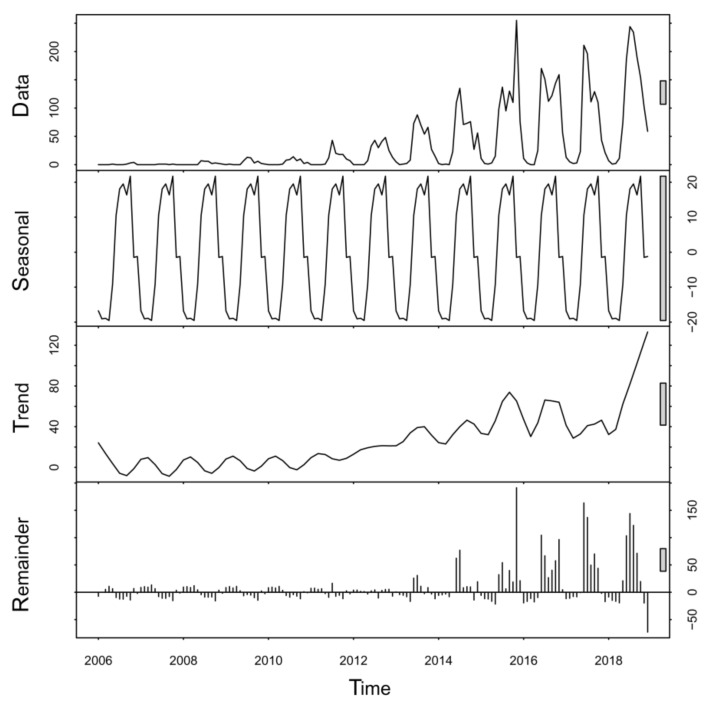
Decomposed scrub typhus cases in the study area from 2006 to 2018.

A seasonal-trend decomposition of time series analysis was performed to decompose the monthly data of ST into the overall trend and the seasonal trend (Figure 2). There was a periodicity vibration in the raw data with one year (12 months) being a cycle, and there was obvious seasonality during the variation process in each year, including two peaks in each cycle. The primary peak was in autumn (September–October), a smaller peak was in summer (June–August), and there were sporadic cases reported in winter-spring (January–April). In addition, the incidence of ST showed an overall upward trend and a periodical increase, but there was a sharp increase in 2018 with 1264 ST cases.

### 3.2. Spatial Distribution Analysis for ST

**Figure 3:**
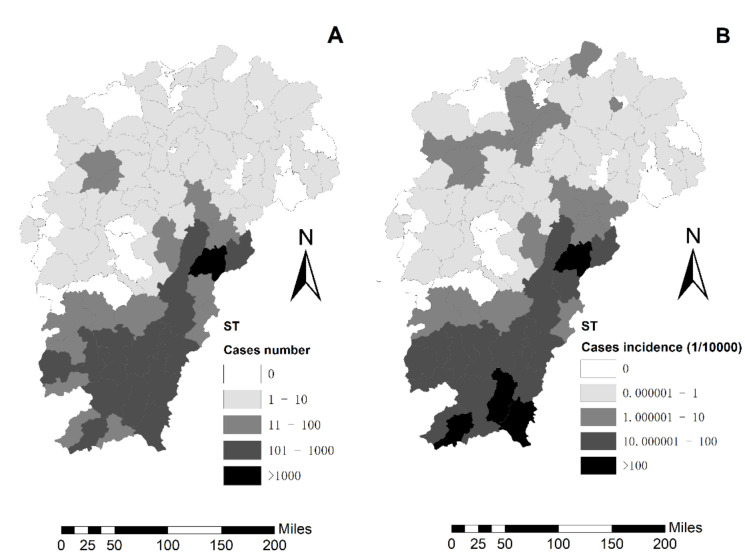
Spatial distribution of scrub typhus cases in the study area. (**A**) Spatial distribution of ST case. (**B**) Cumulative incidence of ST case.

A total of 5508 ST cases were reported and distributed in 79 counties in Jiangxi from 2006 to 2018. According to spatial mapping, the annual cumulative number of ST cases in each county ranged from 0 to 1223. Of these, the counties with more than 100 cases were concentrated in Jiangxi southern mountainous region and Wuyi mountainous hilly region, among which Nanfeng county had the highest (1223 cases); The counties with less than 10 cases were mainly distributed in the plain region bordering on rivers and lakes, Jiangxi’s northern hilly region, and Wugong’s mountainous hilly region (Figure 3A). The top ten counties with the highest ST cumulative incidence Nanfeng (424.75/100,000), Xunwu (189.10/100,000), Anyuan (123.55/100,000), Longnan (122.21/100,000), Xinfeng (77.25/100,000), Lichuan (74.32/100,000), Yihuang (63.38/100,000), Chongyi (55.01/100,000), Nankang (52.94/100,000) and Huichang counties (46.05/100,000) (Figure 3B).

### 3.3. Spatial Autocorrelation Analysis for ST

The results of global spatial autocorrelation revealed that the annual global Moran’s *I* indexes ranged from −0.03 to 0.58 in the whole study period with all the *p*-values less than 0.05 except 2006. This suggested that the spatial distribution of the ST epidemic was significant heterogeneity from 2007 to 2018, with t-shaped spatial autocorrelation characteristics at county-level scale. Additionally, Moran’s *I* indexes presented an uptrend from 2007 to 2013 and a downward trend after 2014, suggesting that the spatial aggregation first increased and then decreased (Table 1).

**Figure 4:**
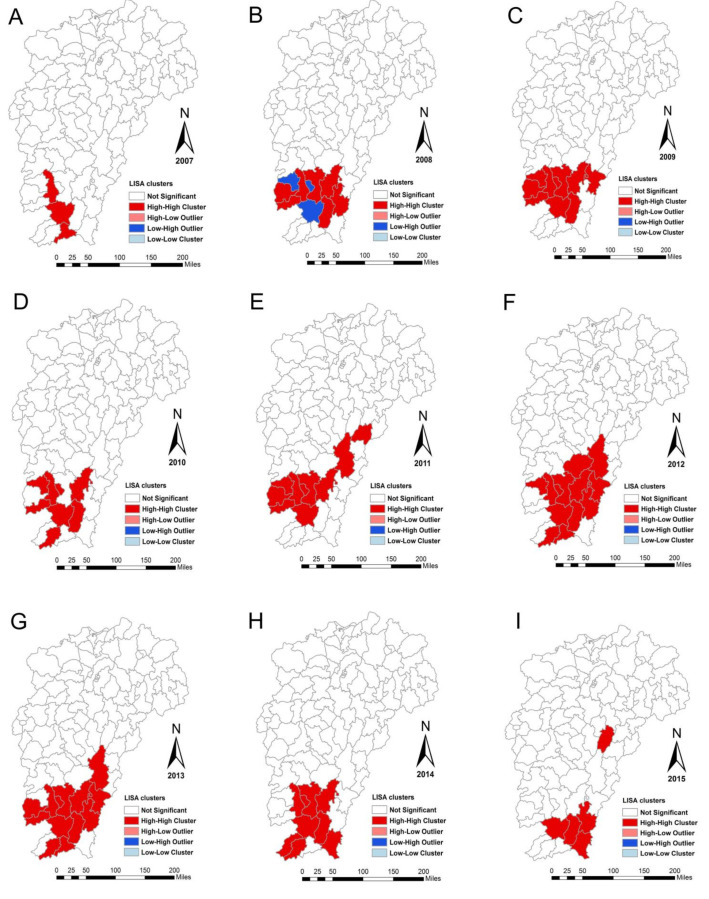

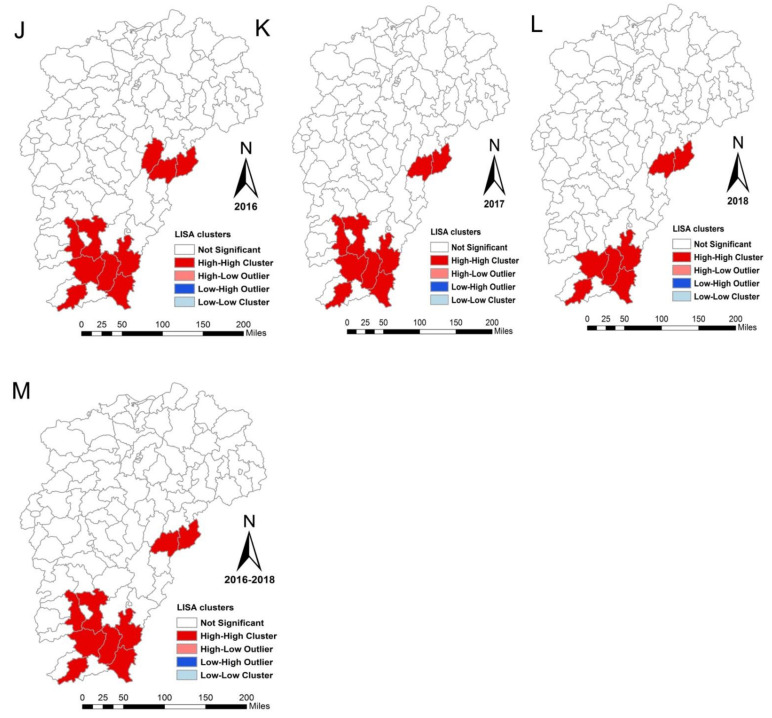
Local indicators of spatial association cluster maps for scrub typhus in the study area from 2007 to 2018. (**A**) 2007, (**B**) 2008, (**C**) 2009, (**D**) 2010, (**E**) 2011, (**F**) 2012, (**G**) 2013, (**H**) 2014, (**I**) 2015, (**J**) 2016, (**K**) 2017, (**L**) 2018, and (**M**) 12 years.

The spatial distribution of ST cases had no significant heterogeneity in 2006, so the LISA was performed to detect the hot spots (High–High cluster area) and outliers of ST transmission in Jiangxi from 2007 to 2018. High–High cluster areas were first identified in Jiangxi southern mountainous region covering Nankang, Xinfeng, and Longnan counties in 2007, and then expanded to surrounding counties and caused the formation of a large, contiguous geographic area of ST incidence. After 2014, two relatively independent and stable High-High cluster areas were identified, one was in Wuyi’s mountainous hilly region as Nanfeng, Yihuang, and Lichuan counties, and the other was in Jiangxi’s southern mountainous region, including Huichang, Xunwu, Anyuan, Ganxian, Nankang, Longnan, and Xinfeng counties. It is worth noting that Nanfeng county was a hot spot in 2011, 2016, 2017, and 2018, not a hot spot from 2012 to 2015. Additionally, the outliers (Low–High cluster area) were also observed in 2008, including Xinfeng, Zhanggong, and Shangyou counties (Figure 4).

### 3.4. Spatiotemporal Clusters Analysis for ST

**Figure 5:**
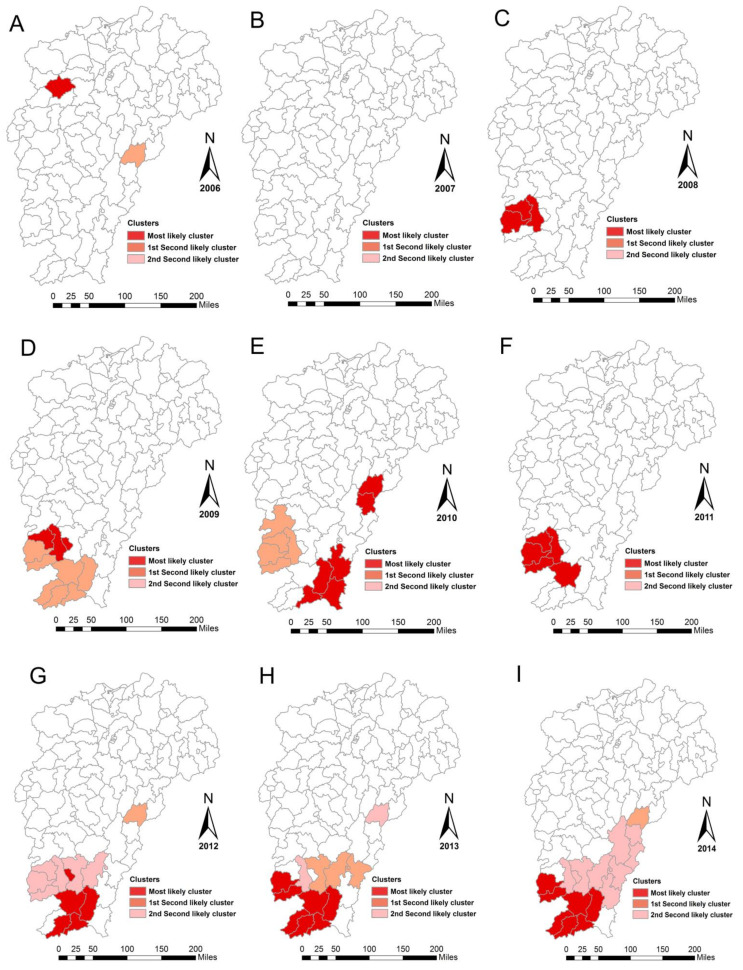

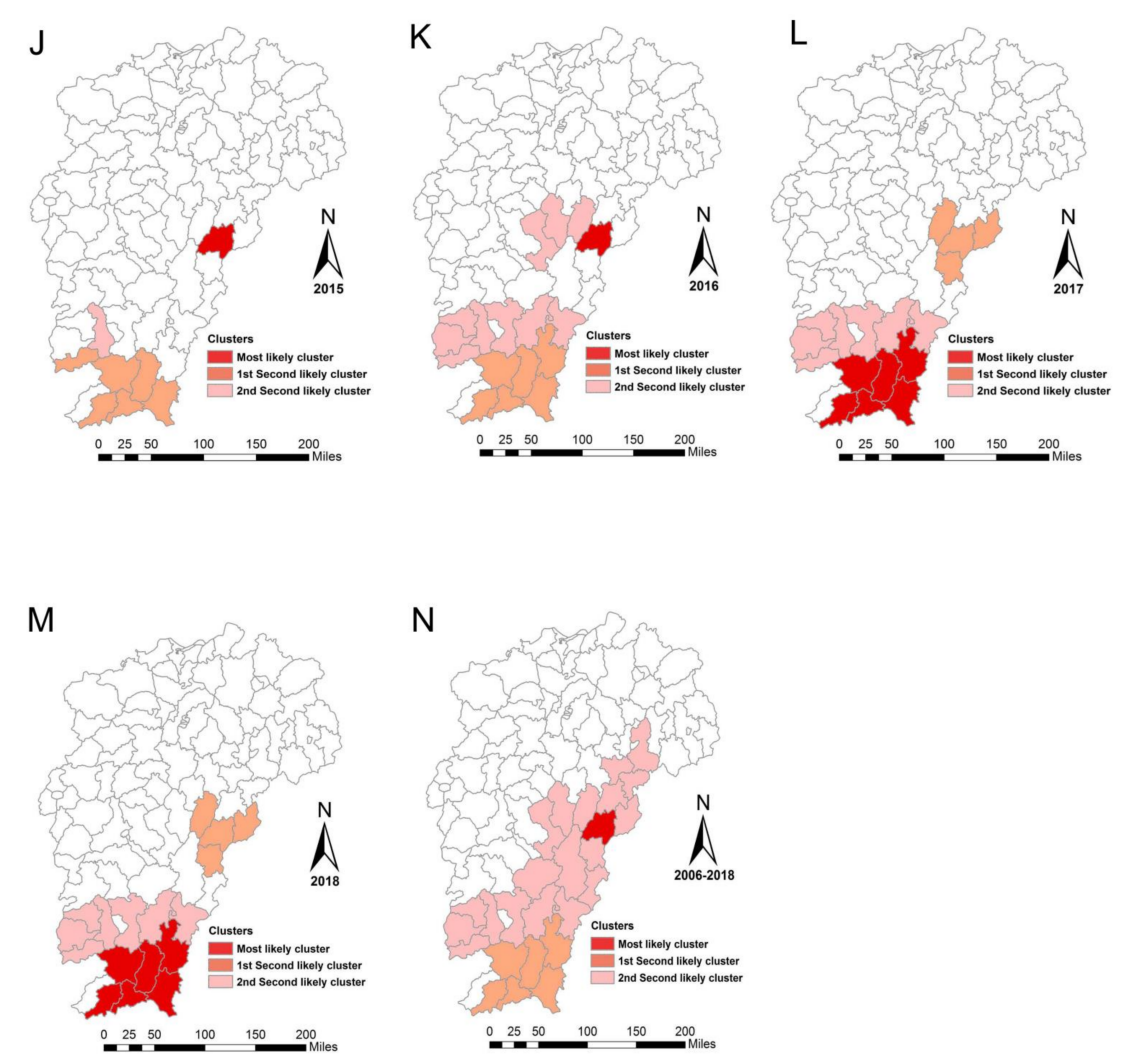
Spatiotemporal clusters of scrub typhus in the study area from 2006 to 2018. (**A**) 2006, (**B**) 2007, (**C**) 2008, (**D**) 2009, (**E**) 2010, (**F**) 2011, (**G**) 2012, (**H**) 2013, (**I**) 2014, (**J**) 2015, (**K**) 2016, (**L**) 2017, (**M**) 2018, and (**N**) 13 years.

The incidence of ST was aggregated through space and time using Kulldorff’s space-time scan statistics. The results revealed that the locations of spatiotemporal clusters changed a lot in the 13-year duration. The most likely cluster was located in Jiangxi’s northern hilly region as Yifeng county in 2006, in Wuyi mountainous hilly region as Nanfeng county in 2010, 2015, 2016, and in Jiangxi southern mountainous region covering 13 counties in the other years. Secondary clusters were mainly located in Jiangxi’s southern mountainous region and Wuyi’s mountainous hilly region, with two to five clusters each year (Figure 5).

Additionally, spatiotemporal clusters across the whole study period from 2006 to 2018 were identified by Kulldorff’s spatiotemporal scan statistic. The most likely cluster only included one county, Nanfeng county. A total of 287,932 human beings were included with a time frame from September 2015 to November 2016. The expected case number was 3.45, while the observed case number was 567. The relative risk for the analysis was 183.10 (LLR = 2359.17, *p* < 0.05). It is worth noting that the most likely cluster accounted for only 0.65% of the total population, while included 10.3% of the total cases during that time. Besides, the first secondary cluster covered six counties in Jiangxi southern mountainous region as Dingnan, Longnan, Xinfeng, Anyuan, Huichang, and Xunwu counties (Table 2). The second secondary cluster covered five counties in Jiangxi’s southern mountainous region as Chongyi, Dayu, Shangyou, Nankang, and Zhanggong counties, thirteen counties in Wuyi’s mountainous hilly region as Zixi, Lichuan, Nancheng, Yihuang, Le’an, Yongfeng, Xingguo, Guangchang, Shicheng, Ningdu, Ganxian, Yudu, and Ruijin counties, and Jinxi and Guixi counties in Jiangxi’s northern hilly region.

## 4. Discussion

The results of this study revealed that the current control and prevention measures in Jiangxi had not been enough to curb the prevalence of ST, which had become a major threat to the health of residents. From 2006 to 2017, the number of ST cases reported in Jiangxi steadily increased, and steeply increased to 1264 cases in 2018. We speculated that there might be an outbreak of ST in some areas of Jiangxi in 2018, resulting in more reported cases than in previous years. For example, an outbreak investigation in Jingjiang city of Jiangsu found that a total of 272 cases were identified between October and December 2013 with the common exposure history of bundling of waste straw, which had been identified as a major risk factor for this ST outbreak [31]. Furthermore, the spatial distribution of ST cases reported in 2006 was not significant heterogeneity, suggesting that these cases might be unrelated and sporadic. Still, one (or a few) strain(s) of *O. tsutsugamushi* might be circulating locally, and there was a risk of ST outbreak. Additionally, this study found that some counties of Jiangxi had developed into the worst affected areas of the ST epidemic, such as Nanfeng county, which only became a hot spot of ST epidemic in 2011, but not a hot spot between 2012 and 2015, and then developed into a stable hot spot after 2016. An explanation could be the emergence of a new *Orientia* species in Nanfeng county in 2011, and the sheer number of people at risk of contracting the disease increased as its foci continued expansion, causing that the number of ST cases reported in Nanfeng county significantly increased after 2016 [32]. Therefore, public health authorities should strengthen routine surveillance of ST, and conduct outbreak investigations in areas with sudden increases in cases.

This study showed that the timing and characteristic of ST outbreaks were remarkably consistent each year, with the high incidence seasons being in summer (June–August) and autumn (September–October), especially in autumn, which is similar to that observed in the southern provinces as Zhejiang [6] and Guangdong [33], but different from that of the northern provinces as Shandong [34] and Jiangsu [35]. A possible explanation was that this diverse seasonal distribution of ST could be attributed to the heterogeneous geographic distribution of the dominant mite in this area. For example, the higher populations of *L. Pallidum* and *L. scutellare* at the same time might lead to the higher incidence of ST from October to December in Korea [36]. Undoubtedly, this seasonal variation will prompt public health departments to allocate resources according to the peak of incidence and the months with more reported cases when formulating ST control policies.

The results of this study showed that the distribution of ST cases had been expanding, covering most counties of Jiangxi, and the high incidence counties were mainly distributed in Jiangxi’s southern mountainous region and Wuyi’s mountainous hilly region. The global model highlighted that the distribution of ST cases in Jiangxi had spatial aggregation from 2007 to 2018. The ISA model and spatiotemporal scan analysis confirmed that the occurrence of ST outbreaks in counties varied year by year, but mainly distributed in Wuyi’s mountainous hilly region as Nanfeng county and Jiangxi’s southern mountainous region as Huichang, Xunwu, Anyuan, Longnan, and Xinfeng counties.

Meanwhile, spatiotemporal scan analysis also demonstrated that the counties at risk of ST outbreak had spread to the surrounding counties, as far as Guixi county in Jiangxi’s northern hilly region and Yongfeng county in Wugong’s mountainous hilly region. Such spatial variation in incidence could be explained by two factors. First, the lack of disease may reflect the absence of the pathogen rather than the lack of a suitable environment for vectors. The counties with the highest incidence were all located in the border areas of Jiangxi, bordering on Guangdong and Fujian. However, since the 1950s, these two provinces have been identified as natural foci of the disease, with a high incidence [18,37]. Furthermore, these counties are mainly mountainous and hilly, with mountains isolated from rivers, forming some deep valleys, complicated topographic landforms, and landscapes with high biodiversity, which likely provide a suitable habitat for chigger mites and rodent hosts [38]. Once infected rodents or chigger mites immigrated to these counties from neighboring Guangdong or Fujian, this suitable habitat likely developed into new foci of infection, leading to a higher incidence in these counties [3,39]. Second, previous studies showed that dry-field farmers were more susceptible to ST than farmers in general, because flood-susceptible chiggers survive poorly in flooded rice paddies [7,40].

Compared with other counties in Jiangxi, more farmers in these counties were engaged in dry fields as fruit planting and picking, because these counties were the leading producer of the Nanfeng orange or the Gannan navel orange [41,42,43]. Moreover, when the farmer worked in the orchards, they frequently used permissible trails on the mountain, which increased the chance of them being exposed to ST. Thus, when people are engaged in activities outdoors, they should adopt several specific protection measures, such as wearing protective clothing, avoiding lying on the grass and using insect repellents.

While this study brings important new knowledge on the epidemiology of ST in Jiangxi, two limitations should be considered when interpreting the findings. Firstly, bias could exist in this study because all the data were extracted from a passive surveillance system. Data quality may be influenced by the availability of diagnostic technology and underreporting. The number of reported cases was much smaller than the actual number of cases, affecting the geographic comparisons of incidence. Moreover, it was not possible to distinguish which strain of *O. tsutsugamushi* was infected by the ST cases. However, the data used in this study were the most comprehensive and reliable data on ST available at national and subnational levels in China. Secondly, the rodent is an important intermediate host for the transmission of ST. However, it was difficult to collect available data on rodents in Jiangxi, as it was impossible to explore the association among human cases, pathogens, hosts, and vectors.

## 5. Conclusions

In conclusion, this study analyzed the spatiotemporal dynamics of ST comprehensively in Jiangxi from 2005 to 2018. This study revealed that a geographical and seasonal variation existed in Jiangxi, and the high-risk areas mainly occurred in Jiangxi’s southern mountainous region and Wuyi’s mountainous hilly region, expanding to the counties of Jiangxi’s northern hilly region. Immediate measures should be taken in high-risk areas, such as health education and awareness promotion, treatment and diagnostic practices, and enhancement of surveillance. We will implement an intensive and extensive survey of chiggers and their hosts in Jiangxi, and explore the relationship between environmental and socioeconomic factors and human morbidity, and further predict the consequence of environmental change for human risks to ST.

## Figures and Tables

**Table 1:** Global spatial autocorrelation analysis of reported scrub typhus in the Jiangxi province of China, 2006–2018.

Year	Moran’s *I*	*Z*-Value	*p*-Value
2006	−0.03	−0.41	>0.10
2007	0.16	3.06	<0.01
2008	0.12	3.87	<0.01
2009	0.27	6.1	<0.001
2010	0.31	5.62	<0.001
2011	0.37	6.92	<0.001
2012	0.51	9.01	<0.001
2013	0.58	10.53	<0.001
2014	0.37	7.05	<0.001
2015	0.07	2.84	<0.01
2016	0.21	4.98	<0.001
2017	0.33	6.04	<0.001
2018	0.24	4.75	<0.001

**Table 2:** Spatiotemporal clusters of scrub typhus detected using Kulldorff’s space-time scan statistic in Jiangxi *, 2006–2018.

Clusters	Longitude	Latitude	Radius (km)	Time Frame	No. Counties	No. Obs	No. Exp	LLR	RR	Incidence (1/100,000)	% Population	% Cases
1	116.49	27.12	0.00 **	2015/9–2015/11	1	567	3.45	2359.17	183.1	196.92	0.65	10.3
2	115.65	24.9	95.07	2017/6–2018/8	6	780	26.49	1939.08	34.13	35.27	5	14.16
3	114.19	25.67	54.05	2017/6–2018/8	4	173	18.23	236.7	9.76	11.37	3.44	3.14
4	115.5	25.93	46.99	2013/6–2014/8	3	134	24.2	120.67	5.65	6.64	4.56	2.43
5	117.03	27.74	54.43	2018/5–2018/10	5	74	7.24	105.62	10.34	4.93	3.39	1.34
6	115.83	27.37	40.52	2017/6–2018/8	3	63	11.96	53.88	5.32	6.31	2.26	1.14
7	116	26.59	59.75	2018/6–2018/9	4	34	6.49	28.88	5.27	1.68	4.58	0.62

* Significant clusters with *p* < 0.01; ** Only 1 county was included in the cluster; Cluster 1: Primary cluster; Clusters 2–7: Secondary clusters; No. Counties: number of counties within clusters; No. Obs: number of observed cases; No. Exp: number of expected cases; LLR: log likelihood ratio; RR: relative risk of the cluster compared with the rest of the country. Incidence: Scrub typhus incidence during the clustering time.

## Data Availability

Correspondence and requests for materials should be addressed to G.L. or H.C. The dataset analyzed is available from the corresponding authors, upon reasonable request.
